# Re-evaluation of the evolution of influenza H1 viruses using direct PCA

**DOI:** 10.1038/s41598-019-55254-z

**Published:** 2019-12-17

**Authors:** Tomokazu Konishi

**Affiliations:** Graduate School of Bioresource Sciences, Akita Prefectural University, Akita, Japan

**Keywords:** Evolutionary genetics, Taxonomy, Immunogenetics, Immunopathogenesis

## Abstract

The history of influenza H1 virus was re-evaluated by applying a new methodology to sequencing data; this objective method enables comparisons among viral types. The approach led to the segregation of all segments of swine and human viruses into three distinct groups: two of them included the pandemic 1977 and 2009 human viruses, and the remaining group may be new in humans. These three groups might have originated from avian viruses and drifted out independently. Genome shifts occurred occasionally among swine viruses; however, distances between avian and swine/human viruses negated the existence of direct shifts from avian viruses. In humans, only one or two viruses appeared each year, which suggests the presence of competition among viruses that migrated freely. All segments drifted continuously under certain rules and constant velocity. Viruses that had caused an outbreak did not appear again over subsequent decades, which may mean populations had become immune to such viruses. In contrast, the viruses in livestock were rather conserved and maintained unique strains in small, separate areas. Such collections of swine strains included human segments, which could become an epidemic in the future.

## Introduction

Although most influenza viruses infect waterfowl exclusively, some of them target humans and livestock with serious symptoms. Some of these viruses cause outbreaks among humans annually, while others decimate chickens in farms and can infect humans fatally^1–4^. There are four types of viruses, A–D; type A is the most virulent and consists of eight segments of the RNA genome, which frequently undergoes mutation during replication, leading to an annual *drift* in viral genomes. Moreover, they may exchange genome segments among different types, causing a *shift* or *reassortment* that produces a new set of viruses, when they infect a same cell^1–3^. As swine can be infected by various viruses, they are considered as a mixing vessel that produces a new virus^3,5^. Vaccines would be useful to prevent outbreaks of this virus^1^. However, this requires an estimation of the types that would cause an outbreak during the following season. To achieve this, we should determine how the viruses have changed.

This article reports the changes that occurred in the influenza H1N1 type from 1977 to 2017, which were analysed using a direct principal component analysis (PCA) method^6^. Compared with the tree-based methods^7^, which have been used to classify viruses for years, this method has a superior objectivity because it does not estimate unfalsifiable assumptions. It can present increased sample data, is able to observe differences of bases and samples coincidently, and the dimensional structure of sequence data is preserved. These characteristics facilitate comparisons of the results among types and segments. The relationships between the samples have become obvious, thus providing accurate classifications.

While tree-based clustering methods has been used in phylogenetics, such methodologies have shortcomings in terms of falsifiability^6^. In particular, they are inappropriate for studying the evolution of human influenza viruses, because they are polyphyletic. They not only change annually but are transferred from other species or emerge from unsuspected regions and can then spread worldwide. Therefore, sometimes they change drastically; here I express the phenomenon as a leap, for avoidance of confusion with a shift, which shows re-assortment of genomes. Such a leap is against the assumptions of tree-shaped models.

## Results

### Classification of viruses

**Figure 1:**
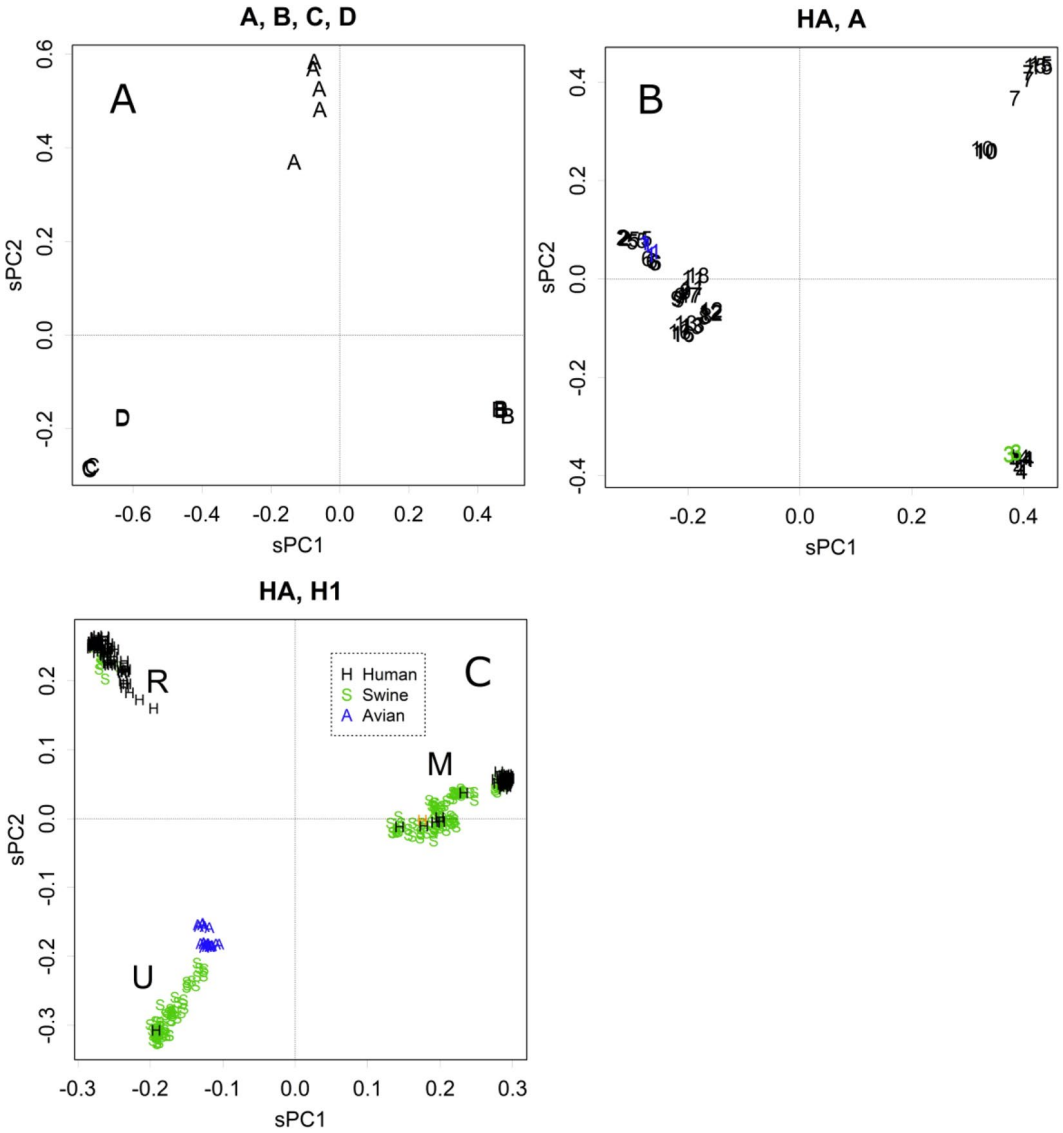
(**A**) PC1 and PC2 of HA, type A–D Influenza viruses, the scaled value (*n = *18). (**B**) HA of the type A virus. The numbers indicate the viral subtypes (*n = *59). (**C**) HA of the H1 subtype (*n* = 387). The M and R groups include the human pandemic 2009 Mexico and 1977 Russia viruses, respectively. Black, human; green, swine, and blue, avian. The orange-coloured “H” in the M group is one of the strains classified as the “triple reassortant”^8^. The H in the U group is the human virus found in Switzerland (NCBI taxonomy ID: 940568).

Let us consider the varieties of the hemagglutinin (HA) gene, which is encoded in an RNA segment, the product of which is the major antigen that appears on the surface of viruses. The method presented here divided the types of viruses into three large groups (Fig. 1A). As the differences among the samples were large, to ease the alignment, amino acid sequences were analysed in Fig. 1. In panel A, the distances among the types were ≥0.5 (Fig. S1A); this value shows the average rate of the difference at any residue between pairs of sequences^6^. The sPC values are the projections of those distances on the PC axes, which concentrate at certain sets of residues (Fig. S1B). Each axis contributes to some part of the differences (Fig. S1C). Viruses were classified based on antigenic differences of other segments; however, the segregation showed likely differences in HA as well. The viruses differed by several indels; in particular, types C and D had extra domains that conferred different activities. Moreover, type A class viruses were further segregated into three groups (Fig. 1B), and the clusters were further classified into lower PCs, showing the characteristics of the subtypes (Fig. S1, Table of PC for samples).

The amino acid residues that contributed to the segregation of HA had certain flexibility; i.e., several choices were possible at the position. Therefore, PC for residues showed segregation into three directions in both cases, as even if a residue showed an extreme value in the PC for residues, the opposed residues would have diffused values. The three clusters did not run through the origin (Fig. S1B,D). If the changes were a choice between the two, opposite directions would yield a point-symmetrical shape (for example, Fig. S1E,F). Separations among types appeared mainly in PC1 and PC2, so the contributions were concentrated on these axes (Fig. S1G).

HA segments of Subtype H1 viruses were further separated into three groups (Fig. 1C). The separation was discrete, with no intermediates. For descriptive purposes, the three groups were termed R, M, and U. The R and M groups included the Russia 1977 and Mexico 2009 pandemic viruses, respectively, and the U group was most likely unfound from recent human records. The avian viruses were located inside the triangle formed by the three groups. Residues that contributed to the separation were a choice between the two, forming a hexagonal shape (Fig. S1E). Differences within a type appeared in low-level PCs; however, swine can accept the wide variations that appeared in PC1 and 2 (Fig. 1C).

The tendency to segregate into the three groups was also observed for the other genome segments (Fig. S2); the R, M, and U groups appeared from the top, clockwise. The groups that include the segments of the two pandemic viruses, Russia 1977 and Mexico 2009, are termed R and M, respectively, with U being the remaining group. As the distances among the groups are similar in all segments, they were placed similarly on the plots (Fig. S2). Variations in the rotation angle of the triangle were caused by differences in the number of samples among the groups. Fig. S2 includes several very old strains: the 1918 and 1943 human pandemic strains (green) and the 1930 swine strains (grey); they showed a tendency to be located closer to the origin of the plot. The magnitudes of PCs were similar among the segments, showing that the groups were separated by similar distances. This suggests a constant mutation rate among the segments, which might be branched from the ancestral strain coincidentally as a new set of viruses. The complete separations observed negate the possibility of homologous recombination between viruses, which would yield some intermediates among the groups. Those of the 1930 swine strain may be an exception among the intermediates, which will be discussed below.

The combinations of segments varied in swine strains (Table 1: PB, RNA polymerase basic protein; PA, RNA polymerase acidic protein; NP, nucleoprotein; NEP, nuclear export protein and non-structural protein). In contrast, in human strains, the combinations were all M or all R.

**Table 1:** Combinations of the segments in swine strains.

Year	District	ID*	Types of the segments							
			PB2	PB1	PA	HA	NP	NA	MP	NEP
1998	US**	1068961	M	M	M	H3	M	N2	U	M
1930	US	380342	R	nd	R	M/R	M/R	U/R	U/R	M/R
1988	US	380341	R	R	R	M	M	U	U	M
1991	US	441597	R	R	R	M	M	U	U	M
2005	US	590637	M	M	M	R	M	R	U	M
2007	US	556272	M	M	M	M	M	U	U	M
2005	US***	398800	M	(R)	M	R	M	R	U	M
2011	US	1166417	M	M	M	M	M	M	M	M
2012	US	1449545	M	M	M	M	M	U	M	M
2011	Mexico	1820554	M	M	M	M	M	M	M	M
2012	Mexico	1820566	R	M	M	M	M	M	M	M
2014	Mexico	1820589	M	M	M	M	M	M	M	M
1985	France	624775	U	U	U	U	U	M	M	U
1992	England	382847	U	U	U	U	U	M	M	U
1993	England	624781	U	U	U	U	U	M	M	U
1993	Denmark	624780	U	U	U	U	U	M	M	U
1998	England	624787	U	U	U	U	U	M	M	U
2003	Spain**	374441	U	U	U	U	U	M	M	U
2005	England	1654110	U	U	U	R	U	N2	M	U
2005	Germany	456883	U	U	U	R	U	N2	M	U
2005	England	1176041	U	U	U	U	U	M	M	U
2010	Switzerland****	940568	U	U	U	U	U	M	M	U
2011	France	1654182	U	U	U	U	U	M	M	U
2012	Netherlands	1654269	U	U	U	U	U	M	M	U
2003	Thailand	519832	U	U	U	M	U	M	M	U
2005	Thailand	488885	U	U	U	M	U	M	M	U
1981	Japan	387253	R	R	R	M	M	U	U	M
2004	China	452465	M	R	R	R	R	R	R	R
2006	China	452464	R	R	R	R	R	R	R	R
2007	China	649625	U	U	U	U	U	M	M	U
2007	China**	568225	M	M	M	M	M	N2	U	M
2012	China	1511996	U	U	U	U	U	M	M	U

*NCBI taxonomy ID.

**Triple reassortant.

***Triple reassortant H1N1, human.

****Detected from human sample.

PB: polymerase basic protein, PA: polymerase acidic protein, HA: hemagglutinin, NP: nucleoprotein, NA: neuraminidase, MP: matrix proteins, NEP: non-structural proteins. R and M are those found in the pandemic 1977 Russia and 2009 Mexico human viruses. U is virtually unfound in humans.

It should be noted that avian viruses showed lower PC values and appeared around the centre of the PCA, while swine and human viruses exhibited extreme values (Figs. 1C and S2). Thus, avian viruses had sequences that were similar to the average among samples at amino acids that were characteristic to the three groups of human and swine viruses: R, M, and U. Avian samples also showed characteristic motifs at other positions, which may appear in lower PCs.

The relationships observed among the strains presented here are different from the classic ones^4,8–12^ in several elements. For example, the number of swine classes was conspicuous. Previously, “triple reassortant”, “Eurasian avian-like,” and “classic” viruses were estimated^8^. However, the triple-reassortant group was absorbed into the two groups, U and M, and the sorting differed among segments in the strains presented here (Fig. S2, blue, Table 1). The frequency of shifts detected was another of the differences detected; i.e., only a few shifts were estimated^4,8,9,13^. These may be derived from differences in the classification methods used, which will be discussed below.

### Only limited positions of a virus can be changed

Although amino acid sequences varied among the types, the three-dimensional (3D) structures of the viruses were rather conserved regarding HA (Fig. S3A), which suggests that at least some positions were conserved. In fact, several positions were common to type A viruses; e.g., they were located at the core of the protein, the rod of the alpha helix (Fig. S3B). These positions seem to be important for the maintenance of the protein functions.

From 1977 to 2009, many residues in the type R viruses changed (Fig. S3C, asterisks). They are mainly located on the surface of the protein, where they may form epitopes. Therefore, the changes that occurred at those positions would be beneficial for escaping the immune system of humans. Conversely, we identified characteristic residues of the R, M, and U groups, which were indicated by the hexagonal directions of PC1 and PC2 (Fig. S1E, green). They exhibited higher scores because they appeared unchanged in only one of the groups (Fig. S3C). These residues might play important roles in the function of the respective protein.

### Annual changes and migration of viruses

#### Human viruses

**Figure 2:**
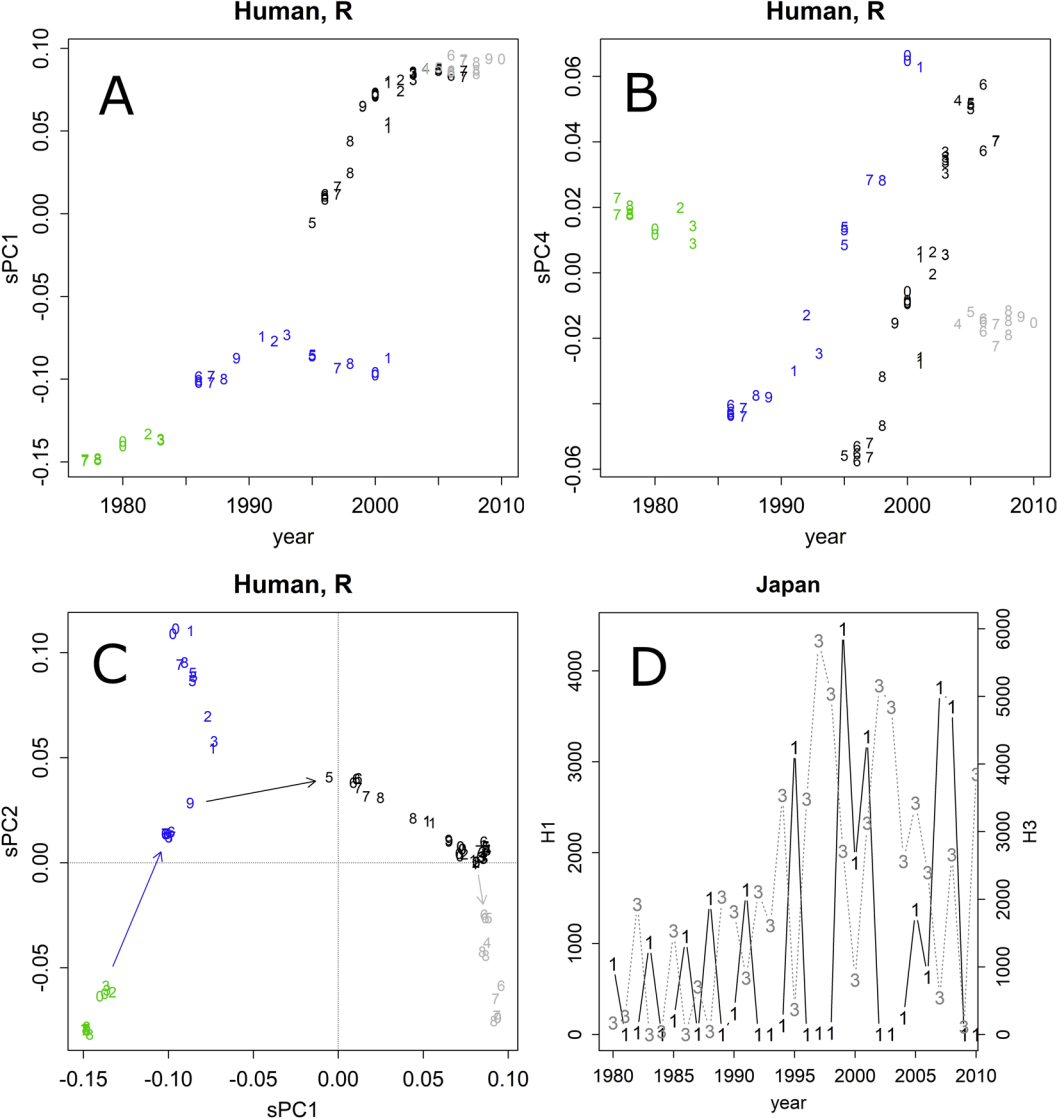
(**A**,**B)** Annual changes of PC1 and PC4, HA of the R group human strains (*n* = 83). The numbers indicate the last digit of the year. The colours indicate the estimated branches of evolution. Other PC axes are presented in Fig. S4C. (**C**) Leaps found in the group R strains. In the last branch (grey), the values did not change in PC1, but they reverted in PC2 after 30 years. The numbers indicate the last two digits of the year. (**D**) Annual changes in H1 (1) and H3 (3) patient counts in Japan.

Let us consider the annual changes of the group R in humans (Figs. 2A–C and S4A). In any given year, only a few variations of the H1 subtypes were found. They were changed slightly by drifting and were detected in another area in the subsequent year (Figs. 2D and S5). As the lives of humans span several decades, they might be immune to viruses that appeared in the past. This may represent a selective pressure to viruses. Moreover, humans may travel to other countries by air while carrying viruses. Hence, the viruses spread worldwide, unifying the outbreaking strains. Although changes in one to several residues may be sufficient to escape specific antibody defenses^1^, humans have various antibodies that target different epitopes. The drifts that occur within a year would be insufficient for viral spread in the same district; hence, H1 outbreaks occurring in two consecutive years were rare (Figs. 2D and S5). Thus, the areas that record an outbreak change yearly (Fig. S5).

The axis of PC1 exhibited contributions from bases that changed once, whereas PC2 was characterized by bases that changed back and forth (Fig. S4A). This oscillation might have been caused by sequential random walk mutation^6^. The random walk model was also supported by the rapid attenuation of the contributions and the accordance with the tangent rule (Fig. S4)^6^. However, a constant pattern of oscillation did not appear in lower-level PCs, suggesting that the changes were not fully random; rather, they seemed to obey some unidentified rules.

As the number of samples is much smaller than the number of bases, the dimension of the PCA is determined by the samples. In particular, as the samples do not differ much within a year, the actual dimension is determined by the total number of years, forming the drops at the end of the contributions; this also bends the tangent regression at its lowest part (Fig. S4).

In addition to the annual continuous drifts, several leaps were also observed (Fig. 2C, arrows between different colours). For example, variants coloured in blue started in 1985, kept drifting, and were recorded through to 2001, going around the world several times. Those coloured in black were found in 1995 and were recorded through to 2007. Those in grey did not change in PC1, but they went back toward the PC2 axis (other directions also appeared in lower PCs, with contributions from positions that changed several times; Fig. S4A). The leaps would refresh the character of the epitopes; e.g., the black leap might allow the parallel evolution of the blue and black variants detected in PC4 (Fig. 2B). The leaps seemed to occur during the years in which the viruses were not detected in the areas that reported the surveillance viral counts and sequences (Figs. 2D and S5).

Leaps were also observed in other segments; although the distance of jumps may have exhibited a certain variation, their timing was similar, and the relationships among the segments that appeared in PC1 and PC2 were all lambda shaped, pointing to the three terminals (Fig. S6). The magnitude of the differences was very similar among the segments, which suggests the presence of a constant mutation rate among the segments.

As was the case for the H1 viruses, the changes seemed to be a choice between two amino acids, each of which showed extreme positive or negative values in PC for bases; therefore, the directions of PCs for residues ran through the origin, showing a symmetrical hexagonal shape in HA (Fig. S1F). The variation of permissive amino acids should be limited at each position.

#### Livestock viruses

**Figure 3:**
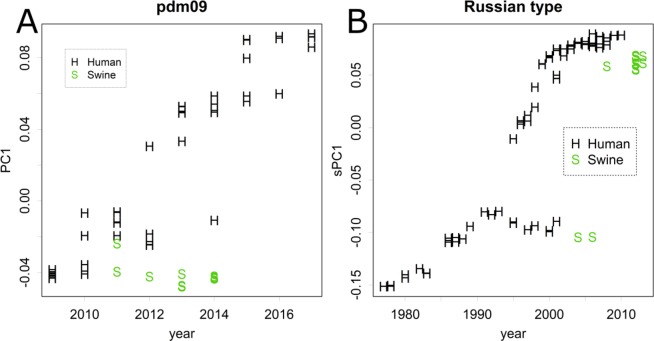
(**A**) HA of the group M viruses found after 2009 (*n* = 73). Black, human; green, swine. Although human viruses showed drifts annually, those of swine remained the same. (**B**) HA of the group R strains (*n* = 98). Those of the swine viruses were found after some years and originated from outbreaks among humans.

Let us consider the annual changes among the viruses of livestock (swine H1: Fig. S4B; chicken H5: Fig. S4C). Many variations were present coincidently in any given year, especially in recent years, when the sequences became available from various areas. Such a variety may hide the effects of drifts from higher axes of the PCA. In fact, the PC values formed horizontal rows in the plots. However, the variations detected in an area did not change much, even in lower PC axes (Fig. S4B,C). The small changes may result from differences in the districts that were surveyed in specific years, rather than from those caused by drifts. Moreover, strains that may be transmitted from humans to swine were detected several years after the human epidemics; this may have occurred if the strains were kept unchanged among swine (Fig. 3A,B). These observations suggest that livestock viruses are rather conserved. Each district may keep its own set of a virus library and we may be observing only a limited portion of the variation. The conserved character of these viruses suggests the presence of a weaker selective pressure for newer epitopes. The variety suggests an absence of competition among viruses; i.e., they might have a limited ability to migrate.

**Figure 4:**
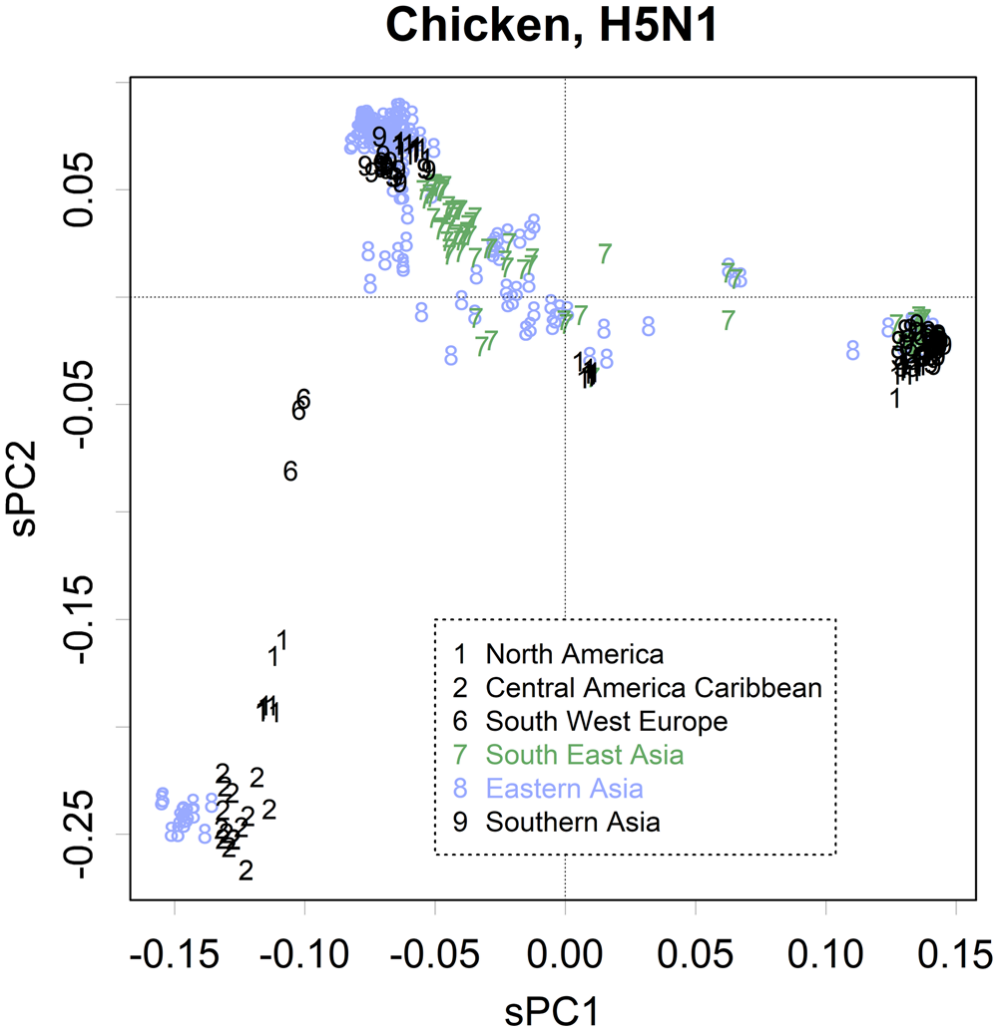
PC1 and PC2 of H5 from chicken (*n* = 485). The numbers indicate the areas identified here.

There were a few exceptions to this conservative endemic rule; e.g., many types of H5N1 chicken viruses were detected in East Asia (Fig. 4). Viruses might be carried by imported livestock. Such migration has also been reported for viruses from swine that were transported from Europe to Mexico^11,12^. The group U swine viruses were further separated into European and Asian subgroups and one of the European viruses was found in Mexico (Fig. S4B; U, PC1, indicated as “2”).

#### Origin of pdm09

The pdm09 virus appeared soon after the last outbreak of type R, which occurred in 2009, and spread worldwide within 1 year (Fig. S5)^3,8,9,11^. The origin of pdm09 is thought to be swine viruses from Mexico^8,9,11^; however, swine strains were not recorded before the pandemic in that country. A later study disclosed that several viral types existed^11^, which included groups that were mainly found in Asia or Europe (Fig. S4B), as well as human M and R viruses (Fig. 3A,B).

Although group M viruses were reported sporadically in humans before 2009, the human pdm09 virus was characteristic as a small cluster in the plot (Fig. 1C). Such characteristics were also observed in the other segments (Fig. S2).

## Discussion

**Figure 5:**
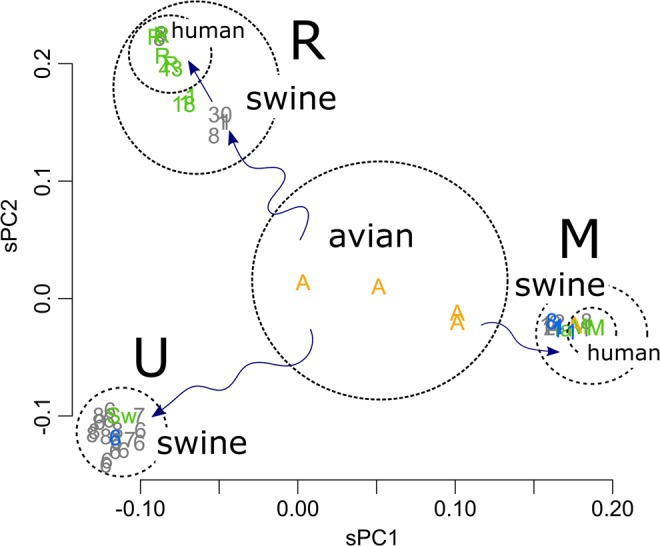
Schematic model of the history of the R, M, and U groups. The origin was avian viruses, which can infect various waterfowl species and exhibit certain variations. Three of them drifted out and were transmitted to swine. They drifted further and caused outbreaks among humans for a while.

The analysis of data for scientific purposes requires objectivity. This cannot be achieved without considering the falsifiability of models^14,15^. Here, we presented views that are very different from those of previous studies. The discrepancies were generated by differences in the methodologies, because many of the data analysed were common among studies. Here, we wish to show that our study has better falsifiability^6^. A sequence matrix is essentially composed of multivariate data with a large number of dimensions^6^. Previous studies used tree-based models (Fig. 1) which can handle only a single dimension^7^; such tree models have to ignore the real data structure. The understanding of clusters requires advanced judgement calls; hence, it may fail easily and the comparison of the results of different segments or types is difficult. In contrast, PCA does not have limitations regarding the shape of presentations or the number of dimensions; thus, it has better objectivity^6^. For instance, the attributions of clusters presented in Figs. 1C and S2 should not cause confusion. Moreover, the assumption that “the relationship among samples should be a tree-like structure” cannot be verified. Our analyses are free from such assumptions. One of the major differences observed was the direct shift from avian to swine or human viruses. Although these shifts were thought to explain the 1918^5,13,16^ and 2009^8,9,11^ pandemic viruses, the segments of avian viruses were separated from any swine or human segments, even those that were thought to be close to the source (Fig. S2, A, orange)^17^. Therefore, it is difficult to estimate such direct shifts. In fact, the cluster called “triple reassortant” disappeared (Fig. 2S); this class was detected as a pandemic swine H3N2 virus^17^ and also presented some clusters of H1N1^8,9,11^. Although avian viruses might be the origin of the H1 viruses, they might have a limited capacity to infect swine or humans, and vice versa. Among the samples studied here, there was only one example of such transmittance, which may have occurred from humans to turkeys (NCBI taxonomy ID: 1848980) in contrast to the occasional transmittance observed between humans and swine (Figs. 1C and 4A,B). The three groups, R, M, and U, may have branched out from the origin to achieve better infectivity in swine or humans (Fig. 5). The 1943 human strain may represent an earlier process of branching out (Fig. S2). Swine may be the origin of the 1918 pandemic virus, i.e., the 1930 swine strain may be a derivative of this group of viruses.

Drifts and spreading: the genomes of the R group of human viruses have been changing yearly (Figs. 2A–C and S4A). The changed residues covered almost the whole surface of the HA protein (Fig. S3C), showing the trajectory of the 30 years of battle against the human immune system. In the last few years, drifting was not observed in PC1, which records positions that changed only once (Fig. 2A, grey). It seems that few positions that were amenable to variation were left unchanged, and that spontaneous mutation failed to hit those positions (Fig. S3C, positions located on the surface of HA and not marked). Even the viruses that almost exhausted the number of unchanged positions kept drifting in the lower PCs, where repeated mutations were recorded (Fig. S4A).

The leaps of drifting observed here (Fig. 2C) may not denote punctuated evolution; rather, they may be the result of accumulating mutations that occurred in areas that do not report sequences. For instance, very few records are available from the African continent. The history of the R type also included some bifurcations (Fig. 2A,C); i.e., viruses located on different branches appeared in different districts in parallel. If these viruses competed within a small area, only the strain with the highest infectivity would remain. If some drifts occurred coincidently in areas located far apart, such branching might have occurred and might have been maintained for a while.

In contrast to the frequent drifts observed in the human strains, those of livestock seemed to be conserved. The livestock strains differed among districts and remained constant for years. The conservative nature of these viral sequences may be attributed to the short life-span of livestock. As they rarely live for a year, they are not immune to viruses that spread over the previous year. Hence, these viruses are not under a selective pressure toward the formation of newer epitopes. Likely, a conservative character was also found in our preliminary studies of human infection with filoviruses and coronaviruses (not shown). Moreover, because livestock rarely move around, the area of viral transmission is limited. For example, even the very infectious pandemic avian virus of 2004 was unable to spread worldwide. Instead, it infected a limited number of farms^2^. This limitation precludes a competition among strains, which enabling the maintenance of unique strains in small, separate areas. Some of the strains that are retained may include segments that spread among humans in the past. It is even possible that several human viruses are kept intact among swine. Such collections may be the source of the 1977 pandemic virus, which is thought to be a frozen laboratory strain that was released accidentally^4,18^.

The migration of livestock viruses could be mainly caused by trading^11^, although migrant birds might be able to transport avian viruses to chickens in some cases^19^. However, if such transfers occur frequently, the characteristics of specific areas would disappear, and viruses would be better distributed worldwide; in particular, Europe should have viruses that originated in other areas (Fig. S2). Therefore, viruses of livestock would be transmitted rarely by migrant birds.

We should be aware of the risk of keeping groups of swine that have different influenza strains in adjacent farms because new viruses may be produced by genome shifting. To prevent this, the segregation of imported swine from domestic ones is critical. Districts that report several viral types should prevent farm-to-farm contamination. Moreover, the exchange of genomes between human and swine viruses may give rise to a new strain with new sets of surface proteins that is able to infect humans. The Switzerland virus (NCBI taxonomy ID: 940568) proved that the group U strain is also capable of infecting humans; therefore, a new pandemic may occur in the case of an outbreak of this type of virus. Such viruses of human origin are maintained among swine, maybe latently (Fig. 3A,B).

Origins of the segments: the pdm09 virus may have originated via shifts among swine viruses^8,10,11^. The American classic virus and the reassortant virus could be defined using the segments (R, R, R, M, M, U, U, M) and (M, M, M, M, M, U, U, M) from segments 1 to 8, respectively (Table 1). Replacing the U groups of NA and MP with those of the European type (U, U, U, U, U, M, M, U) can complete all M segments of pdm09. Such shifts may occur occasionally (Table 1).

It should be noted that assortment of the M segments was insufficient to produce the pdm09 virus. Although the reassortant virus was repeatedly detected in human samples, it was transmitted from swine and did not spread among humans. The segments showed PCs located nearer the average compared with pdm09 (Figs. 1C and S2). If a human-to-human transition requires the specific characteristics of a virus, those characteristics should be detected in some of the PCs when enough samples are included in the calculation and the extreme values observed in PC1 and PC2 may reflect such characteristics. The archiving of the characteristics requires further shifts in all the segments of the assorted M virus.

The origin of M type polymerases in the reassortant and pdm09 viruses may not be the result of a direct shift from avian viruses (Fig. S2). Instead, they might be maintained among swine and may have drifted away from the avian type (Fig. 5). The same would be true for the R type segments. Although most of the sequencing data were obtained from humans, the R type may have been overlooked among swine. If these viruses do not cause severe symptoms, they may not attract the attention of farmers or researchers. In fact, focused approaches detected the R type in swine (Fig. 3B)^11^. Such endemic R types may show lower values in PC closer to the origin; e.g., those found in England and Dötlingen in 2005 might satisfy this condition (Fig. S2; HA), and other segments also exhibited such endemic candidates.

Unfortunately, it is difficult to estimate the history of old viruses because of the shortage of data. Some of the segments of the 1930 strain exhibited interesting positions on the branches: i.e., some were R type, while others were located between the R and (M or U) types (Fig. S2). The segments would be those close to the branching point of the groups. However, this does not necessarily mean that the branching occurred recently; i.e., it could have been preserved among swine at least until 1930. Whether the 1918 and/or 1943 human strains are lineal ancestors of the 1977 pandemic virus remains uncertain. It should be noted that these viruses were included in different epidemic series on different memories of immunity among humans. These immune memories are changing because of the change of human generations, i.e., the return of the R group observed over recent years (Fig. 2C, grey) might have been enabled by such changes.

Estimation of future viruses and vaccination: patterns of change were observed for the human R and M types in each of the PC axes (Figs. 2A,B, 3A, and S4A). Although PCA produces sin curves for the records of random walk mutations^6^, the real patterns may reflect some rules in the drift of genomes. As patterns of M groups become apparent, we can compare them between M and R viruses to estimate the hidden rules.

Although HA is thought to be the primary target of the human adaptive immune response^1^, other segments that include proteins located inside the virus particle also drifted with a magnitude similar to that of HA, which suggests a common mutation frequency (Fig. S4C). This observation was unexpected because mutations may lead to malfunction; if they are not the target of immunity, they should be conserved, similar to those of livestock viruses. As the segments are displayed on the surface of infected cells, they will activate specific immune cells, which may play important roles in the human immune system.

It is obvious that any strain that has caused an outbreak will not appear again for decades among humans. In contrast to the case of the H3N2 viruses, H1N1 outbreaks rarely occur in two consecutive seasons (Figs. 2D and S5). This shows that most people exposed to the virus are infected asymptomatically during the viral outbreak and become immune; otherwise, the virus could infect a portion of receptive people that were not infected in the previous year. Also, the drift occurring in a single year is insufficient for escaping the immune system. The pdm09 virus appeared after the flu season and spread worldwide from April 2009 to March 2010 (Fig. S5). The extraordinary infectivity of this virus may have been caused by epitopes that were new to humans. Therefore, the present system of vaccine production to control influenza infections, which uses eggs, has a critical defect: i.e., none of the stock strains that are used for viral production are the one that will cause an outbreak in the following season. The population might have become immune to the stock viruses and the vaccine just helps to recall the memory of these strains.

## Materials and Methods

### Sequence data analysis

All of the sequence data used here were obtained from the NCBI database (https://www.ncbi.nlm.nih.gov/nuccore). Data pertaining to amino acid sequences were used to observe multiple subtypes or hosts (Figs. 1 and 2), while nucleotide sequences were used for specific subtypes (Figs. 3–5, S5 and S6). Data alignment was performed using MUSCLE^20^. All the aligned sequences are presented in Figs S1, S2 and S4. The sequence matrix was casted to direct Principal Component Analysis (PCA)^6^, which analyses sequence matrix without losing any of original information in the matrix^6^, using R^21^. The direct PCA is resistant to differences caused by the alignment: differences in the conditions or the alignment methods may not alter the results practically^6^. The sequence matrix were translated into a Boolean vector **X**; for *n* samples of *l* bases, **X** is a matrix of *n* × 5 *l*, which correspond to the nucleotides and inserted space “-“ for alignment. A mean vector $\overrightarrow{{\rm{m}}}$ was estimated as $\overrightarrow{{\rm{m}}}=\mathop{\sum }\limits_{i=1}^{n}{{\bf{X}}}_{i,j}/n$, where *j* = 1, 2,,,, 5 *l*. The boolean vector was then catered to estimate differences to the mean vector, ${{\bf{D}}}_{k,}={{\bf{X}}}_{k,}-\overrightarrow{{\rm{m}}}$, where *k* = 1, 2,,,, *n*. Then **D** was subjected for singular value decomposition as ${\bf{D}}={\bf{L}}{\boldsymbol{\Sigma }}{{\bf{R}}}^{\ast }$. Scaled values of the Principal Components (sPC) were found by placing back the singular values ${\boldsymbol{\Sigma }}$ to the unitary matrixes **L** or **R**; those for the samples were estimated as ${{\rm{sPC}}}_{{\rm{s}}}={\bf{L}}{\boldsymbol{\Sigma }}/\sqrt{l}$and the nucleotide bases were estimated as ${{\rm{sPC}}}_{{\rm{n}}}={\bf{R}}{\boldsymbol{\Sigma }}/\sqrt{n}$. They were scaled for comparisons that enables to identify which base contributed for the differences of samples^22^. Therefore, each sPC presents directions and distances of the differences, which were recorded by the unitary matrix and the singular value, respectively, as sets of orthogonal vectors executed as the columns of the sPC matrixes. Scripts of the R used for the calculation is presented in Fig. S2. PC signs were derived in a random manner; thus, the results were adjusted to align different presentations for comparison. A starter kit for the direct PCA is available in the accompanying article^6^.

### The 3D structures

The PDB files were obtained from the RCSB Protein Data Bank (https://www.rcsb.org/). Position data were found using ATOM header: i.e., positions at the alpha carbon of amino acids were connected to draw the structure and were then aligned using PCA. The HTML5 output was generated using the writeWebGL function of the *rgl* package of R (https://cran.r-project.org/web/packages/rgl/vignettes/rgl.html).

## Supplementary information

data set 1
