# Supplementary material for: Re-evaluation of the evolution of influenza H1 viruses using direct PCA
Source: Sci Rep. 2019 Dec 17;9:19287. doi: 10.1038/s41598-019-55254-z (PMC6917806; doi:10.1038/s41598-019-55254-z)
Supplement: Supplementary file 1 — data set 1 [file 41598_2019_55254_MOESM1_ESM.zip › information/supplement.html]

Supple


## Supplementary Information

for **Re-evaluation of the evolution of influenza H1 viruses using direct PCA**  
  
Tomokazu Konishi

## Figures

and related data with R codes.

|  |  |
| --- | --- |
| S1 | Classification of Influenza viruses |
| S2 | Segments of H1N1 subtypes. |
| S3 | The 3D structure of hemagglutinin. |
| S4 | Annual changes in Human H1N1, group R. |
| S5 | Influenza Surveillance in the selected areas. |
| S6 | Human A H1N1, group R. |

  
