# Supplementary material for: Re-evaluation of the evolution of influenza H1 viruses using direct PCA
Source: Sci Rep. 2019 Dec 17;9:19287. doi: 10.1038/s41598-019-55254-z (PMC6917806; doi:10.1038/s41598-019-55254-z)
Supplement: Supplementary file 1 — data set 1 [file 41598_2019_55254_MOESM1_ESM.zip › information/supplement/S1.html]

S1


## Figure S1

Click images to enlarge

| S1A | 1A | S1B | S1C |
| --- | --- | --- | --- |
| Types A-D, Distance | PC for sample | PC for residues | Contribution for the types |
|  |  |  |  |
|  |
| S1D | S1E | S1F | S1G |
| PC for residues, Type A | Subtype H1 | Group R | Contribution for H1 |
|  |  |  |  |

  

**Fig. S1.**  Histogram of the distances among types of viruses (**S1A**).   
The values are expectations that a site (base or residue) is different between the pairs of samples.   
If the value is 1, all the sites will be different.   
The distances are then projected to the PC axes (Fig. 1A).   
Types B and C differed by a value of 0.5 in sPC1; thus, the rate of similarity between sites is 50% on average.   
In contrast, they were almost identical in sPC2; thus, the sites are identical.   
In this manner, each of the axes observes certain sets of residues, which appear in the PC for residues (**S1B**).   
A dot indicates a residue; the values (and the residue) can be checked in a table.   
The axes are sorted by the magnitude of the projected distances,   
showing the contribution of each site (**C** and **G** for the cases of Fig. 1A and 1B, respectively).   
**D**, **E**, and **F**: PC for residues for Fig. 1 A, 1B, and 1C, respectively.

  

## Sequences

Fig. 1A & S1A-C  Sequences of types A-D viruses  
Fig. 1B & S1D  Type A viruses  
  
 R code for amino acid sequences

## Table of PC for Samples

Type A samples (Fig. 1B)
