# Supplementary material for: Re-evaluation of the evolution of influenza H1 viruses using direct PCA
Source: Sci Rep. 2019 Dec 17;9:19287. doi: 10.1038/s41598-019-55254-z (PMC6917806; doi:10.1038/s41598-019-55254-z)
Supplement: Supplementary file 1 — data set 1 [file 41598_2019_55254_MOESM1_ESM.zip › information/supplement/S1/PC_Atype.html]

S1


## PC for sample of H1 subtypes

| HA | PC1 | PC2 | PC3 | PC4 | PC5 | PC6 | PC7 | PC8 | PC9 | PC10 | PC11 | PC12 | PC13 | PC14 | PC15 | PC16 | PC17 |
| --- | --- | --- | --- | --- | --- | --- | --- | --- | --- | --- | --- | --- | --- | --- | --- | --- | --- |
| H1/AF091317.1 | -0.190918831 | 0.05 | -0.16 | 0.03 | -0.01 | 0.06 | -0.04 | 0.08 | -0.09 | 0.30 | -0.01 | 0.01 | 0.00 | 0.00 | -0.02 | 0.01 | -0.03 || H1/EU382985.1 | -0.190918831 | 0.04 | -0.15 | 0.03 | 0.00 | 0.06 | -0.04 | 0.06 | -0.09 | 0.26 | -0.01 | 0.01 | -0.03 | 0.01 | -0.04 | 0.01 | 0.03 || H1/CY084825.2 | -0.183847763 | 0.04 | -0.14 | 0.04 | -0.01 | 0.06 | -0.04 | 0.07 | -0.11 | 0.29 | -0.01 | 0.02 | 0.00 | -0.01 | -0.01 | 0.02 | -0.03 || H1/KX978381.1 | -0.197989899 | 0.05 | -0.18 | 0.04 | -0.01 | 0.06 | -0.04 | 0.07 | -0.10 | 0.29 | 0.00 | 0.01 | -0.01 | 0.01 | -0.02 | 0.01 | -0.01 || H2/NC\_007374.1 | -0.219203102 | 0.06 | -0.24 | 0.03 | 0.13 | 0.07 | 0.02 | -0.04 | 0.04 | -0.06 | -0.07 | -0.06 | 0.04 | 0.08 | 0.02 | -0.13 | 0.01 || H2/L11138.1 | -0.22627417 | 0.06 | -0.24 | 0.03 | 0.16 | 0.09 | 0.04 | -0.03 | 0.06 | -0.08 | -0.06 | -0.05 | 0.04 | 0.09 | 0.03 | -0.14 | 0.01 || H2/CY206822 | -0.22627417 | 0.06 | -0.25 | 0.02 | 0.16 | 0.09 | 0.04 | -0.04 | 0.05 | -0.08 | -0.07 | -0.04 | 0.04 | 0.09 | 0.02 | -0.14 | 0.01 || H2/KY272859 | -0.22627417 | 0.06 | -0.25 | 0.02 | 0.15 | 0.09 | 0.04 | -0.03 | 0.06 | -0.08 | -0.07 | -0.04 | 0.04 | 0.09 | 0.03 | -0.13 | 0.01 || H3/NC\_007366.1 | 0.275771645 | -0.25 | -0.08 | 0.01 | 0.04 | -0.05 | -0.33 | -0.04 | 0.02 | -0.02 | -0.02 | 0.01 | 0.01 | 0.01 | 0.01 | 0.00 | 0.00 || H3/AF382324.1 | 0.275771645 | -0.25 | -0.09 | 0.01 | 0.04 | -0.04 | -0.30 | -0.04 | 0.01 | -0.02 | -0.01 | 0.01 | 0.01 | 0.00 | 0.01 | 0.01 | -0.01 || H3/KY284550 | 0.261629509 | -0.25 | -0.10 | 0.01 | 0.05 | -0.06 | -0.33 | -0.03 | 0.02 | -0.01 | -0.02 | 0.00 | -0.01 | 0.00 | 0.01 | 0.00 | -0.01 || H3/KY211004 | 0.261629509 | -0.25 | -0.09 | 0.01 | 0.04 | -0.05 | -0.33 | -0.03 | 0.02 | -0.01 | -0.01 | 0.00 | -0.01 | 0.00 | 0.01 | 0.00 | -0.01 || H4/AF290436.1 | 0.275771645 | -0.26 | -0.06 | -0.01 | -0.01 | 0.04 | 0.18 | 0.02 | 0.01 | 0.03 | -0.02 | 0.09 | 0.11 | -0.06 | 0.12 | 0.01 | -0.01 || H4/KT338365 | 0.282842712 | -0.28 | -0.08 | -0.01 | -0.01 | 0.05 | 0.19 | 0.02 | 0.02 | 0.03 | -0.01 | 0.08 | 0.10 | -0.05 | 0.10 | 0.00 | -0.01 || H4/KX162615 | 0.275771645 | -0.27 | -0.07 | -0.01 | -0.01 | 0.05 | 0.18 | 0.02 | 0.01 | 0.03 | -0.02 | 0.09 | 0.11 | -0.06 | 0.11 | 0.01 | 0.00 || H4/KY284424 | 0.282842712 | -0.28 | -0.08 | -0.01 | -0.01 | 0.04 | 0.19 | 0.01 | 0.02 | 0.03 | -0.01 | 0.09 | 0.10 | -0.05 | 0.10 | 0.00 | -0.01 || H5/AF303057.1 | -0.212132034 | 0.06 | -0.22 | 0.04 | 0.08 | 0.06 | 0.03 | -0.07 | 0.04 | -0.10 | 0.04 | 0.04 | -0.07 | -0.11 | -0.01 | 0.16 | 0.00 || H5/DQ256383.1 | -0.190918831 | 0.06 | -0.21 | 0.02 | 0.08 | 0.05 | 0.01 | -0.06 | 0.05 | -0.08 | 0.05 | 0.04 | -0.06 | -0.10 | -0.01 | 0.18 | -0.01 || H5/KY284403 | -0.212132034 | 0.06 | -0.22 | 0.03 | 0.08 | 0.06 | 0.02 | -0.06 | 0.05 | -0.11 | 0.05 | 0.04 | -0.07 | -0.10 | -0.01 | 0.16 | -0.02 || H5/LC198528 | -0.197989899 | 0.06 | -0.19 | 0.03 | 0.09 | 0.06 | 0.02 | -0.06 | 0.04 | -0.09 | 0.04 | 0.04 | -0.06 | -0.11 | -0.02 | 0.16 | 0.00 || H6/AF474029.1 | -0.183847763 | 0.02 | -0.10 | -0.06 | -0.38 | -0.04 | -0.02 | -0.03 | 0.03 | -0.06 | -0.06 | 0.00 | 0.01 | 0.01 | 0.01 | 0.00 | 0.01 || H6/AF474035.1 | -0.183847763 | 0.02 | -0.10 | -0.06 | -0.37 | -0.04 | -0.02 | -0.03 | 0.03 | -0.06 | -0.05 | 0.01 | 0.02 | 0.01 | 0.01 | -0.01 | 0.01 || H6/KX979411 | -0.190918831 | 0.03 | -0.11 | -0.06 | -0.37 | -0.04 | -0.03 | -0.02 | 0.02 | -0.06 | -0.05 | 0.00 | 0.00 | 0.01 | 0.02 | -0.01 | 0.00 || H6/KY284505 | -0.183847763 | 0.03 | -0.11 | -0.06 | -0.37 | -0.04 | -0.03 | -0.03 | 0.02 | -0.06 | -0.05 | 0.00 | 0.01 | 0.01 | 0.01 | -0.01 | 0.00 || H7/U20462.1 | 0.28991378 | 0.29 | 0.04 | 0.01 | 0.00 | -0.01 | 0.00 | -0.01 | 0.08 | 0.01 | -0.06 | 0.05 | 0.02 | -0.16 | -0.13 | -0.10 | -0.01 || H7/L43915.1 | 0.28991378 | 0.30 | 0.04 | 0.01 | -0.01 | -0.01 | 0.00 | 0.00 | 0.08 | 0.01 | -0.06 | 0.05 | 0.03 | -0.16 | -0.14 | -0.11 | -0.01 || H7/L43914.1 | 0.28991378 | 0.30 | 0.04 | 0.01 | 0.00 | -0.01 | 0.01 | 0.00 | 0.08 | 0.01 | -0.06 | 0.05 | 0.03 | -0.16 | -0.14 | -0.11 | -0.01 || H7/CY035946 | 0.268700577 | 0.26 | 0.02 | 0.01 | 0.00 | -0.04 | -0.01 | 0.01 | 0.06 | 0.03 | -0.03 | 0.01 | 0.01 | -0.07 | -0.07 | -0.02 | 0.02 || H8/CY166784 | -0.120208153 | -0.06 | 0.16 | -0.21 | 0.03 | -0.01 | 0.01 | -0.11 | -0.08 | 0.04 | 0.04 | -0.08 | -0.21 | -0.16 | 0.17 | -0.14 | 0.00 || H8/LC029898 | -0.120208153 | -0.05 | 0.16 | -0.21 | 0.03 | -0.01 | 0.02 | -0.12 | -0.07 | 0.04 | 0.05 | -0.08 | -0.21 | -0.16 | 0.17 | -0.14 | -0.01 || H9/NC\_004908.1 | -0.141421356 | -0.01 | 0.18 | -0.25 | 0.05 | 0.08 | -0.04 | 0.23 | 0.10 | -0.04 | -0.01 | 0.03 | 0.00 | 0.02 | -0.01 | 0.03 | 0.01 || H9/AY206679.1 | -0.155563492 | -0.03 | 0.18 | -0.26 | 0.04 | 0.08 | -0.05 | 0.24 | 0.09 | -0.04 | 0.01 | 0.01 | 0.00 | 0.02 | -0.01 | 0.02 | -0.01 || H9/AY206671.1 | -0.155563492 | -0.03 | 0.18 | -0.25 | 0.04 | 0.08 | -0.04 | 0.24 | 0.09 | -0.04 | 0.00 | 0.01 | 0.00 | 0.02 | -0.01 | 0.02 | 0.00 || H9/AF461531.1 | -0.148492424 | -0.02 | 0.19 | -0.24 | 0.04 | 0.08 | -0.04 | 0.23 | 0.10 | -0.04 | -0.01 | 0.01 | 0.00 | 0.01 | 0.00 | 0.04 | 0.01 || H10/CY186284 | 0.233345238 | 0.18 | 0.01 | 0.00 | 0.00 | 0.09 | -0.01 | 0.08 | -0.34 | -0.15 | -0.01 | 0.01 | 0.00 | 0.04 | 0.02 | 0.02 | 0.01 || H10/CY166394 | 0.233345238 | 0.19 | 0.01 | 0.00 | 0.00 | 0.08 | -0.01 | 0.07 | -0.32 | -0.14 | -0.01 | 0.02 | 0.00 | 0.02 | 0.01 | 0.02 | 0.01 || H10/KX979437 | 0.233345238 | 0.18 | 0.01 | 0.00 | -0.01 | 0.10 | -0.01 | 0.08 | -0.34 | -0.15 | -0.01 | 0.01 | 0.00 | 0.04 | 0.02 | 0.01 | 0.01 || H11/AB450451 | -0.134350288 | -0.01 | 0.06 | 0.09 | -0.06 | 0.07 | -0.05 | -0.02 | 0.00 | -0.02 | 0.37 | -0.04 | 0.14 | -0.01 | -0.03 | -0.08 | 0.02 || H11/HM059993 | -0.134350288 | 0.00 | 0.06 | 0.08 | -0.05 | 0.06 | -0.05 | -0.02 | 0.00 | -0.02 | 0.38 | -0.01 | 0.15 | -0.02 | -0.04 | -0.08 | 0.01 || H12/CY144707 | -0.113137085 | -0.04 | 0.22 | -0.25 | 0.06 | 0.00 | 0.02 | -0.26 | -0.06 | 0.05 | -0.04 | 0.03 | 0.09 | 0.07 | -0.08 | 0.05 | 0.00 || H12/CY190019 | -0.113137085 | -0.05 | 0.22 | -0.25 | 0.06 | 0.00 | 0.02 | -0.26 | -0.06 | 0.05 | -0.04 | 0.03 | 0.09 | 0.07 | -0.09 | 0.05 | 0.00 || H12/KY284499 | -0.113137085 | -0.05 | 0.22 | -0.25 | 0.06 | -0.01 | 0.03 | -0.26 | -0.06 | 0.04 | -0.04 | 0.03 | 0.09 | 0.07 | -0.08 | 0.06 | 0.01 || H13/KX978812.1 | -0.134350288 | -0.06 | 0.25 | 0.27 | -0.01 | 0.06 | 0.00 | -0.02 | 0.01 | -0.03 | -0.04 | -0.01 | -0.01 | 0.04 | 0.01 | -0.01 | -0.16 || H13/KX978076.1 | -0.141421356 | -0.06 | 0.26 | 0.27 | -0.01 | 0.05 | 0.00 | 0.00 | 0.01 | -0.01 | -0.05 | -0.01 | -0.02 | 0.01 | 0.01 | 0.01 | -0.19 || H13/CY239408.1 | -0.141421356 | -0.07 | 0.26 | 0.27 | -0.01 | 0.05 | 0.01 | 0.00 | 0.02 | -0.03 | -0.04 | -0.01 | -0.02 | 0.02 | 0.01 | 0.00 | -0.21 || H14/CY167267.1 | 0.282842712 | -0.25 | -0.06 | 0.01 | -0.04 | 0.03 | 0.16 | 0.02 | 0.01 | 0.00 | 0.04 | -0.13 | -0.14 | 0.08 | -0.16 | 0.00 | 0.01 || H14/JN696316.2 | 0.275771645 | -0.25 | -0.06 | 0.00 | -0.05 | 0.03 | 0.16 | 0.02 | 0.01 | 0.00 | 0.04 | -0.13 | -0.14 | 0.08 | -0.16 | 0.00 | 0.01 || H14/KJ195676.1 | 0.282842712 | -0.26 | -0.06 | 0.00 | -0.04 | 0.04 | 0.16 | 0.02 | 0.01 | 0.00 | 0.05 | -0.13 | -0.15 | 0.08 | -0.16 | 0.00 | 0.01 || H15/L43917.1 | 0.304055916 | 0.31 | 0.05 | 0.01 | 0.00 | -0.03 | 0.01 | -0.04 | 0.11 | 0.06 | 0.04 | -0.05 | -0.02 | 0.11 | 0.11 | 0.06 | 0.01 || H15/L43916.1 | 0.304055916 | 0.30 | 0.04 | 0.01 | 0.00 | -0.04 | 0.01 | -0.04 | 0.12 | 0.06 | 0.04 | -0.06 | -0.03 | 0.11 | 0.13 | 0.07 | 0.01 || H15/CY006009.1 | 0.304055916 | 0.30 | 0.04 | 0.01 | 0.00 | -0.04 | 0.01 | -0.04 | 0.12 | 0.06 | 0.04 | -0.06 | -0.03 | 0.11 | 0.13 | 0.07 | 0.01 || H15/CY098540.1 | 0.296984848 | 0.30 | 0.05 | 0.01 | -0.01 | -0.03 | 0.01 | -0.04 | 0.11 | 0.06 | 0.04 | -0.05 | -0.01 | 0.09 | 0.10 | 0.06 | 0.01 || H16/HM059998 | -0.148492424 | -0.08 | 0.28 | 0.31 | 0.00 | 0.04 | -0.01 | -0.01 | 0.02 | 0.01 | -0.08 | 0.01 | -0.02 | -0.01 | 0.01 | 0.01 | 0.17 || H16/KX977730.1 | -0.148492424 | -0.08 | 0.27 | 0.30 | 0.00 | 0.04 | -0.01 | -0.01 | 0.01 | 0.00 | -0.07 | 0.02 | -0.01 | -0.01 | 0.01 | 0.01 | 0.17 || H16/EU030976.1 | -0.155563492 | -0.08 | 0.26 | 0.30 | -0.01 | 0.04 | 0.00 | -0.01 | 0.02 | 0.00 | -0.06 | 0.02 | -0.02 | -0.02 | 0.00 | 0.01 | 0.17 || H17/CY103892 | -0.141421356 | -0.02 | -0.01 | 0.02 | 0.08 | -0.37 | 0.06 | 0.10 | -0.07 | -0.01 | -0.04 | -0.24 | 0.14 | -0.08 | 0.00 | 0.06 | 0.00 || H17/CY103884.1 | -0.141421356 | -0.02 | -0.01 | 0.02 | 0.08 | -0.37 | 0.06 | 0.10 | -0.07 | -0.01 | -0.04 | -0.23 | 0.14 | -0.08 | 0.01 | 0.06 | -0.01 || H18/CY125945 | -0.127279221 | 0.01 | -0.02 | 0.04 | 0.07 | -0.38 | 0.07 | 0.05 | -0.02 | -0.03 | 0.07 | 0.25 | -0.11 | 0.11 | -0.02 | -0.06 | 0.00 || H18/KR077932.1 | -0.127279221 | 0.01 | -0.02 | 0.04 | 0.07 | -0.38 | 0.07 | 0.05 | -0.02 | -0.03 | 0.07 | 0.25 | -0.11 | 0.11 | -0.02 | -0.06 | 0.00 |

  

## contribution

  
