# Supplementary material for: Re-evaluation of the evolution of influenza H1 viruses using direct PCA
Source: Sci Rep. 2019 Dec 17;9:19287. doi: 10.1038/s41598-019-55254-z (PMC6917806; doi:10.1038/s41598-019-55254-z)
Supplement: Supplementary file 1 — data set 1 [file 41598_2019_55254_MOESM1_ESM.zip › information/supplement/S2.html]

S2


Click images to enlarge

## PC1 vs PC2

Fig. 2S. Segments of H1N1 subtypes. Nucleotide sequences were aligned and analyzed. Green: human strains. Numbers indicates the area of detection: 1, North America; 2, Central America and Caribbean; 6, South West Europe; 7, South-East Asia; 8, Eastern Asia; A, avian virus; M and R, human strains; S, virus infected to human. The aligned sequences and accession numbers are also presented.

|  |  |  |  |
| --- | --- | --- | --- |
| PB2 | PB1 | PA | HA |
|  |  |  |  |
| NP | NA | MP | NEP |  |
|  |  |  |  |  |

  

Abbreviations: R, type R of human; M, type M of human; Sw, the human virus txid940568; Ia; the human triple reasortant virus txid398800. Blue, triple reassortant; Grey, swine; Green, human; A, abian.

  

## Nucleotides sequences

- PB2
- PB1
- PA
- HA
- NP
- NA
- MP
- NEP

## sPC for samples

- PB2
- PB1
- PA
- HA
- NP
- NA
- MP
- NEP

## R script for the calculation

- the script for nucleotide sequences
- test sample

## Contribution

|  |  |  |  |
| --- | --- | --- | --- |
| PB2 | PB1 | PA | HA |
|  |  |  |  |
| NP | NA | MP | NEP |  |
|  |  |  |  |  |

  
