# Supplementary material for: Re-evaluation of the evolution of influenza H1 viruses using direct PCA
Source: Sci Rep. 2019 Dec 17;9:19287. doi: 10.1038/s41598-019-55254-z (PMC6917806; doi:10.1038/s41598-019-55254-z)
Supplement: Supplementary file 1 — data set 1 [file 41598_2019_55254_MOESM1_ESM.zip › information/supplement/S3.html]

S3


## The 3D structure

- **S3A.** H1, H2, H3, and H5
- **S3B.** Positions common to type A
- **S3C.** Positions changed from 1977 to 2009
