# Supplementary material for: Re-evaluation of the evolution of influenza H1 viruses using direct PCA
Source: Sci Rep. 2019 Dec 17;9:19287. doi: 10.1038/s41598-019-55254-z (PMC6917806; doi:10.1038/s41598-019-55254-z)
Supplement: Supplementary file 1 — data set 1 [file 41598_2019_55254_MOESM1_ESM.zip › information/supplement/S4/human/PC_years_Matrix/index.html]

MP


## MP\_year

  
  
  
  
  
  
  
  
  
  
  

## Contribution

  
  

## Nucleotide sequences

MP, human
