# Supplementary material for: Re-evaluation of the evolution of influenza H1 viruses using direct PCA
Source: Sci Rep. 2019 Dec 17;9:19287. doi: 10.1038/s41598-019-55254-z (PMC6917806; doi:10.1038/s41598-019-55254-z)
Supplement: Supplementary file 1 — data set 1 [file 41598_2019_55254_MOESM1_ESM.zip › information/supplement/S4/S4B.html]

Chicken


# Annual changes in HA, Chicken H5N1

Click images to enlarge

|  |  |  |  |  |  |  |
| --- | --- | --- | --- | --- | --- | --- |
| PC1 | PC2 | PC3 | PC4 | PC5 | PC6 | contribution |
|  |  |  |  |  |  |  |

  

## Amino acid sequences

Chicken H5N1 

## PC1 vs PC2

|  |  |
| --- | --- |
| sample | base |
|  |  |

  
