# Supplementary material for: Re-evaluation of the evolution of influenza H1 viruses using direct PCA
Source: Sci Rep. 2019 Dec 17;9:19287. doi: 10.1038/s41598-019-55254-z (PMC6917806; doi:10.1038/s41598-019-55254-z)
Supplement: Supplementary file 1 — data set 1 [file 41598_2019_55254_MOESM1_ESM.zip › information/supplement/S4/S4C.html]

Human


# Annual changes in Human H1N1, R

Click images to enlarge

## Segments

- PB2
- PB1
- PA
- HA
- NP
- NA
- MP
- NEP

## Nucleotides sequences

- PB2
- PB1
- PA
- HA
- NP
- NA
- MP
- NEP
