# Supplementary material for: Re-evaluation of the evolution of influenza H1 viruses using direct PCA
Source: Sci Rep. 2019 Dec 17;9:19287. doi: 10.1038/s41598-019-55254-z (PMC6917806; doi:10.1038/s41598-019-55254-z)
Supplement: Supplementary file 1 — data set 1 [file 41598_2019_55254_MOESM1_ESM.zip › information/supplement/S5.html]

S5


## Influenza Surveillance in the selected areas

### viral species

- H1
- H1pmd09
- H3
- Btotal
- Bvictoria
- Byamagata
- Btotal
- Total
- H1+H3+B
- H1pdm09+H3+B
- BY+BV+B
- Total (raw count data)
