# Supplementary material for: Re-evaluation of the evolution of influenza H1 viruses using direct PCA
Source: Sci Rep. 2019 Dec 17;9:19287. doi: 10.1038/s41598-019-55254-z (PMC6917806; doi:10.1038/s41598-019-55254-z)
Supplement: Supplementary file 1 — data set 1 [file 41598_2019_55254_MOESM1_ESM.zip › information/supplement/S6.html]

S6


Click images to enlarge

# Data simulation for PCA

|  |  |  |  |  |
| --- | --- | --- | --- | --- |
|  |  |  |  |  |
|  |  |  |  |  |

  

## R codes

a<-rbinom(n=1000, size=1, prob = 1/2)   
a[a==0] <- -1   
   
tdata <- array(0, dim=c(200, 1000))   
   
for(i in 1:200){   
position<-sample(1:1000, 2)   
a[position] <- -1\* a[position]   
tdata[i,] <- a }   
   
means<- apply(tdata, 2, mean) # means for each position   
diffs<-sweep(tdata, 2, means) # centering   
  
res\_svd <- svd(diffs)   
   
Left <- res\_svd$u # the left singular vectors   
Right <- res\_svd$v # the right singular vectors   
sqL <- diag(res\_svd$d) # diagonal matrix of the singular values   
  
### calculatinf of pc's   
sPC\_sample <- tdata %\*% Right/1000^0.5   
sPC\_nuc <- t(diffs) %\*% Left/200^0.5   
   
  
dir.create("S6")   
for(i in 1:10){   
png(width=1000, height=1100, pointsize = 40, file=paste("S6/PC", i, ".png", sep=""))   
par(lwd=2, mex=0.6, mai=c(2,2,2,0.2))   
plot(1:200, sPC\_sample [,i], type="l", xlab="round", ylab=paste("sPC", i, sep=""))   
dev.off()}
