# Supplementary material for: Re-evaluation of the evolution of influenza H1 viruses using direct PCA
Source: Sci Rep. 2019 Dec 17;9:19287. doi: 10.1038/s41598-019-55254-z (PMC6917806; doi:10.1038/s41598-019-55254-z)
Supplement: Supplementary file 1 — data set 1 [file 41598_2019_55254_MOESM1_ESM.zip › information/supplement/S7.html]

S6


Click images to enlarge

# Human A H1N1, group R

## PC1 vs PC2

|  |  |  |  |  |
| --- | --- | --- | --- | --- |
| PB2 | PB1 | PA | HA |  |
|  |  |  |  |  |
| NP | NA | MP | NEP |  |
|  |  |  |  |  |
